# A Rare Case of Enlarged Dissecting Aneurysm Occurring One Year After Conservative Management of Azygos Anterior Cerebral Artery Dissection

**DOI:** 10.7759/cureus.70123

**Published:** 2024-09-24

**Authors:** Kenu Ryo, Hidetoshi Mochida

**Affiliations:** 1 Neurological Surgery, Asahi General Hospital, Asahi, JPN

**Keywords:** aneurysm, anterior cerebral artery, arterial dissection, infarction, subarachnoid hemorrhage

## Abstract

Anterior cerebral artery (ACA) dissection is generally managed with conservative treatment, often resulting in a favorable prognosis. However, cases with delayed enlargement of dissecting aneurysms following conservative therapy are rare. We describe a 46-year-old male patient who presented with concurrent subarachnoid hemorrhage and cerebral infarction caused by ACA dissection. Initial digital subtraction angiography (DSA) revealed an azygos ACA, as well as dilation and stenosis in the A2 segment. Follow-up DSA on the sixth and fifteenth days detected dissecting aneurysms at the bifurcation of the left pericallosal artery and at the peripheral bifurcation of the middle internal frontal artery, with intervening stenosis. The patient recovered well without rebleeding after conservative management. However, one year later, magnetic resonance imaging (MRI) indicated an enlargement of the aneurysm. Simple coil embolization was performed, and a 6-month postoperative MRI confirmed the disappearance of the aneurysm. This case suggests that while conservative management of azygos ACA dissections can be effective, careful and long-term follow-up is crucial due to the potential for delayed aneurysm formation.

## Introduction

Dissection of the anterior cerebral artery (ACA) is uncommon, comprising approximately 5% of intracranial artery dissections; however, it represents an important etiology of stroke in young and middle-aged adults [1]. ACA dissection may culminate in infarction, subarachnoid hemorrhage (SAH), or their concomitant occurrence. In a recent meta-analysis of 80 cases, infarction was documented in 73%, SAH in 10%, and co-occurrence in 17% [2]. Most patients underwent conservative management, including the administration of intravenous alteplase, exclusive use of antiplatelet agents, solitary application of anticoagulants, and concurrent use of both antiplatelet and anticoagulant therapies, but without the use of any antithrombotic medication. Interventional or surgical treatment was administered in seven documented cases presenting with SAH or the confluence of ischemia and SAH. Surgical procedures were conducted in four cases, encompassing techniques such as wrapping, trapping, and clipping to address the emergence of a newly developed dissecting aneurysm [2]. Additionally, treatment with coil embolization with and without a stent has been reported [3,4]. Nonetheless, the optimal management of ACA dissection remains debatable. Many patients who undergo conservative treatment achieve favorable clinical outcomes. However, although long-term complications associated with conservative treatment may occur, information regarding them is limited.

Azygos ACA is a rare anatomical anomaly characterized by the formation of a singular A2 segment, with an incidence of 1.16% in 57 of 4913 cases taken for close examination of headache, dizziness, convulsions, stroke, etc. [5]. Distal ACA aneurysms are often observed in cases of azygos ACA. However, there have been no reports of azygos ACA dissection.

Herein, we present the first case of azygos ACA dissection accompanied by delayed enlargement of a dissecting aneurysm after one year of conservative treatment.

## Case presentation

**Figure 1:**
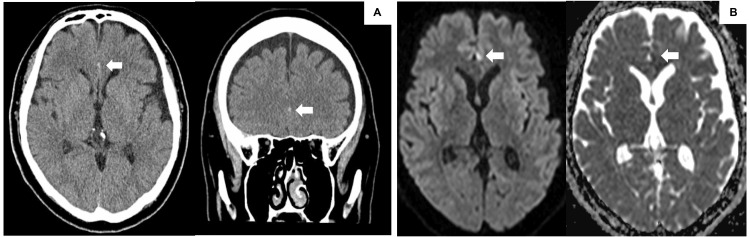
Images at onset of our patient at admission to our department. (A) Computed tomography scan on admission reveals subarachnoid hemorrhage in the interhemispheric fissure (arrows). (B) Magnetic resonance imaging (diffusion-weighted image and apparent diffusion coefficient map image) on admission revealing acute cerebral infarction in the left cingulate gyrus (arrows).

A 46-year-old man was admitted to the neurosurgery department of our hospital in November 2021 with acute loss of consciousness, transient lower-limb weakness, and headache. His preexisting medical conditions included obesity and hypertension. A computed tomography scan revealed an SAH in the interhemispheric fissure, and magnetic resonance imaging (MRI) revealed acute cerebral infarction (CI) in the left cingulate gyrus (Figure 1).

**Figure 2:**
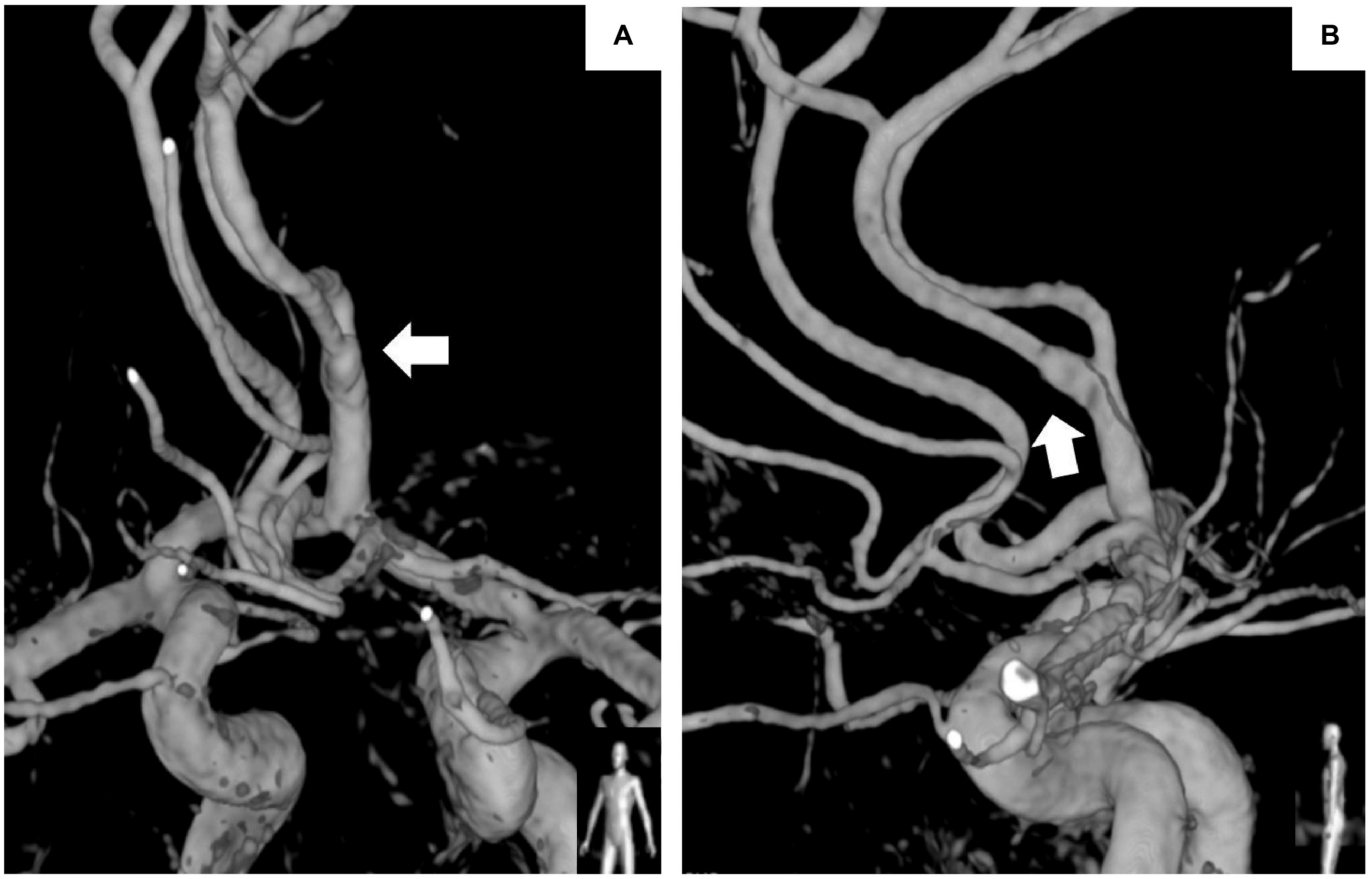
Digital subtraction angiography on admission showing the azygos ACA and an irregular dilatation in the origin of the left pericallosal artery (arrow). (A) Frontal view, (B) lateral view. ACA: Anterior cerebral artery

Digital subtraction angiography (DSA) showed the presence of an azygos artery along with dilation and stenosis in the A2 segment (Figure 2). No antithrombotic medication was administered owing to apprehensions regarding bleeding diathesis and meticulous follow-up was conducted.

**Figure 3:**
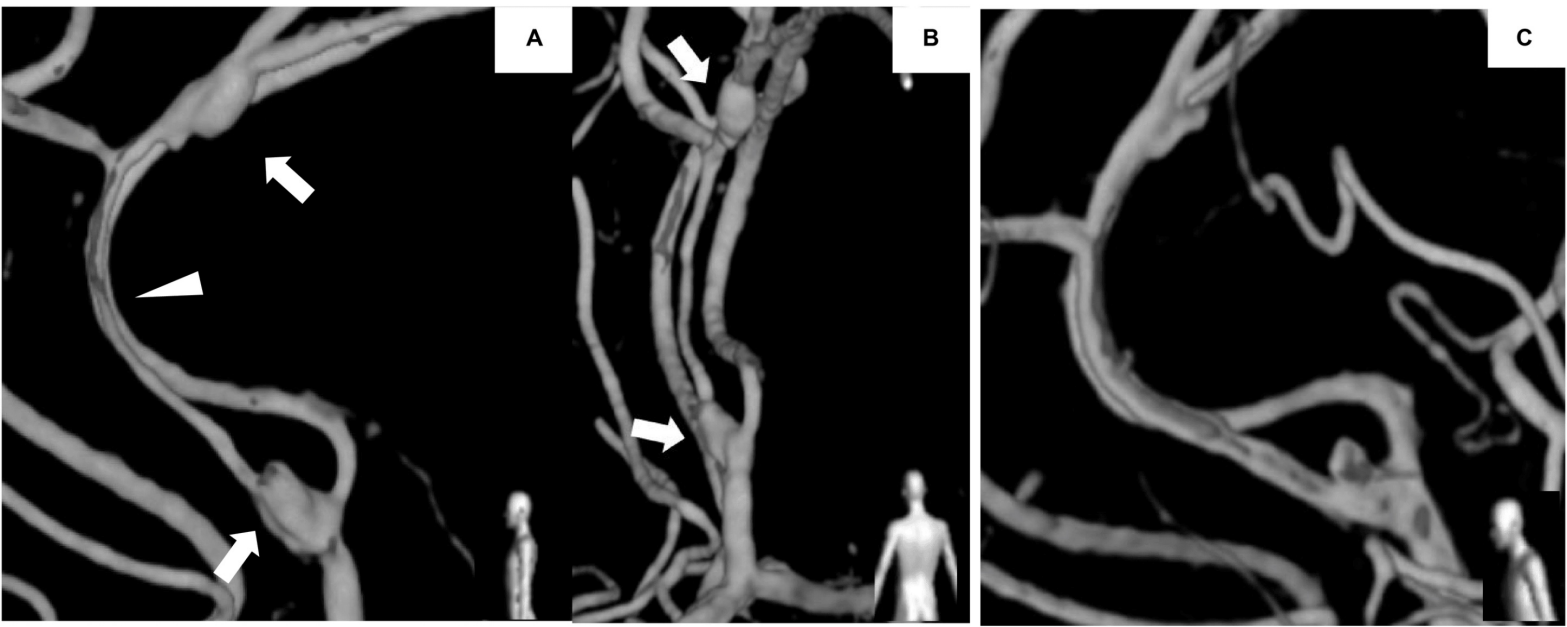
Morphological changes in dissecting vessels. (A, B) Digital subtraction angiography on day 15 showing dissecting aneurysms at the bifurcation of the left pericallosal artery and the peripheral bifurcation of the middle internal frontal artery (arrows), with stenosis between them (arrowhead); (A) lateral view, (B) posterior-anterior view. (C) Digital subtraction angiography on day 43 revealing spontaneous regression of stenosis and peripheral dissecting aneurysm; lateral view.

On the fourth day, the right hemiplegia worsened, and a subsequent MRI revealed an enlarged ischemic lesion. Follow-up DSA performed on the sixth and fifteenth days showed dissecting aneurysms at the bifurcation of the left pericallosal artery and peripheral bifurcation of the middle internal frontal artery, with stenosis between them (Figure 3). DSA performed on day 43 revealed spontaneous regression of the stenosis and a peripheral dissecting aneurysm (Figure 3). The patient remained free from rebleeding and hemiplegia improved, resulting in the patient attaining a modified Rankin scale score of 1.

**Figure 4:**
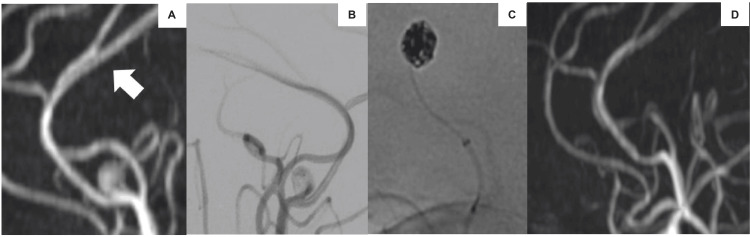
Re-enlargement of aneurysm. (A) MRI one year after onset reveals enlargement of the aneurysm of the left pericallosal artery and disappearance of the other aneurysm (arrow). (B, C) Coil embolization was performed. (D) MRI six months postoperatively. (A), (B), and (D) - lateral view.

Follow-up MRI conducted every three months showed no changes. However, at the one-year follow-up, MRI revealed enlargement of the dissecting aneurysm at the origin of the left pericallosal artery (Figure 4). Simple coil embolization was performed, and a six-month postoperative MRI confirmed the disappearance of the aneurysm. The patient had no remarkable family history or physical findings suggestive of a genetic syndrome. Therefore, genetic testing, including analysis of collagen gene mutations, was not performed.

## Discussion

This case report highlights two important issues. First, aneurysms resulting from azygos ACA dissection may exhibit delayed enlargement. Cerebral artery dissection occurs owing to disruption of the internal elastic lamina and media, and when the dissection extends immediately beneath the adventitia, it can lead to the formation and subsequent rupture of a pseudoaneurysm. Studies conducted on pathological specimens have revealed that cerebral artery dissection undergoes a spontaneous healing process following its onset, which is characterized by complete circumferential coverage of the neointima [6,7]. A case series of 42 patients with ruptured vertebral artery dissection demonstrated that 24 (80%) of 30 re-rupture cases occurred within one week of the initial onset, with the longest duration until re-rupture being 41 days [8]. In another study examining 143 cases of intracranial artery dissection, among 35 with recurrent bleeding, the longest duration until rebleeding was 26 days [7]. Thus, the healing process extends beyond one month, with an estimated completion time of approximately two months. During this reparative phase, dissecting aneurysms and alterations in the shape of the dissected vessels may occur. To the best of our knowledge, there have been no reports of delayed (occurring after two months) aneurysmal enlargement following ACA dissection, the present case being the first. In cases of azygos ACA, the frequency of aneurysms is high, ranging from 7% to 71% [9-12]. Hemodynamic stress exerted on the vessel wall, specifically at the curvature of the corpus callosum genu, is believed to be a contributing factor, along with the presence of a congenital anomalous artery. Thus, the presence of an azygos ACA may have contributed to delayed enlargement of the dissecting aneurysm observed in our case.

Second, conservative management may be safe and effective for patients presenting with simultaneous SAH and acute CI attributable to ACA dissection. In a series of 18 ACA dissection cases, nine were ischemic, five were hemorrhagic, and four exhibited a combination of both [2]. Among the five hemorrhagic patients, three with lesions in the A1 segment received surgical treatment, of whom two were in a vegetative state. The remaining two hemorrhagic patients had lesions in the A2 or A3 segments and were managed conservatively; both patients demonstrated favorable recovery. Among the four combination cases, three were managed conservatively and one received operative wrapping of the lesion. These patients did not experience rebleeding and achieved good recovery. Similarly, in our patient presenting with simultaneous SAH and CI involving a lesion located between the A2 and A3 segments, conservative treatment resulted in a favorable prognosis.

## Conclusions

Conservative treatment may be safe and effective for patients with simultaneous SAH and CI attributable to ACA dissection. Following conservative treatment, meticulous follow-up is crucial considering the potential for delayed aneurysm enlargement. However, the involvement of the azygos artery in delayed aneurysm enlargement remains uncertain. Therefore, further investigations are required to explore the frequency and etiology of late-onset dissecting aneurysms.
